# Placental CX3CL1 is Deregulated by Angiotensin II and Contributes to a Pro-Inflammatory Trophoblast-Monocyte Interaction

**DOI:** 10.3390/ijms20030641

**Published:** 2019-02-02

**Authors:** Olivia Nonn, Jacqueline Güttler, Désirée Forstner, Sabine Maninger, Julianna Zadora, András Balogh, Alina Frolova, Andreas Glasner, Florian Herse, Martin Gauster

**Affiliations:** 1 Division of Cell Biology, Histology and Embryology, Gottfried Schatz Research Centre for Cell Signaling, Metabolism and Ageing, Medical University of Graz, 8010 Graz, Austria; olivia.nonn@medunigraz.at (O.N.); jacqueline.serbin@medunigraz.at (J.G.); desiree.forstner@medunigraz.at (D.F.); sabine.maninger@medunigraz.at (S.M.); 2 Experimental and Clinical Research Center, A Joint Cooperation Between the Charité Medical Faculty and the Max-Delbrueck Center for Molecular Medicine, 13125 Berlin, Germany; Julia.Zadora@mdc-berlin.de (J.Z.); andras.balogh@mdc-berlin.de (A.B.); florian.herse@charite.de (F.H.); 3 Max-Delbrueck Center for Molecular Medicine in the Helmholtz Association, 13125 Berlin, Germany; 4 Berlin Institute of Health (BIH), 13125 Berlin, Germany; 5 Institute of Molecular Biology and Genetic of National Academy of Sciences of Ukraine, 03680 Kyiv, Ukraine; fshodan@gmail.com or a.o.frolova@imbg.org.ua; 6 Femina Med Center, 8010 Graz, Austria; office@dr-glasner.at

**Keywords:** first trimester pregnancy, placenta, angiotensin, CX3CL1, inflammation

## Abstract

CX3CL1, which is a chemokine involved in many aspects of human pregnancy, is a membrane-bound chemokine shed into circulation as a soluble isoform. Placental CX3CL1 is induced by inflammatory cytokines and is upregulated in severe early-onset preeclampsia. In this study, the hypothesis was addressed whether angiotensin II can deregulate placental CX3CL1 expression, and whether CX3CL1 can promote a pro-inflammatory status of monocytes. qPCR analysis of human placenta samples (*n* = 45) showed stable expression of CX3CL1 and the angiotensin II receptor AGTR1 throughout the first trimester, but did not show a correlation between both or any influence of maternal age, BMI, and gestational age. Angiotensin II incubation of placental explants transiently deregulated CX3CL1 expression, while the angiotensin II receptor antagonist candesartan reversed this effect. Overexpression of recombinant human CX3CL1 in SGHPL-4 trophoblasts increased adhesion of THP-1 monocytes and significantly increased IL8, CCL19, and CCL13 in co-cultures with human primary monocytes. Incubation of primary monocytes with CX3CL1 and subsequent global transcriptome analysis of CD16^+^ subsets revealed 81 upregulated genes, including clusterin, lipocalin-2, and the leptin receptor. Aldosterone synthase, osteopontin, and cortisone reductase were some of the 66 downregulated genes present. These data suggest that maternal angiotensin II levels influence placental CX3CL1 expression, which, in turn, can affect monocyte to trophoblast adhesion. Release of placental CX3CL1 could promote the pro-inflammatory status of the CD16^+^ subset of maternal monocytes.

## 1. Introduction

The human placenta functions as an interface between the mother and the fetus by fulfilling a wide-ranging panel of pregnancy maintaining functions, including exchange of gases and metabolites, regulation of water balance, dissipation of heat, and secretion of endocrine and immune-modulating factors. The vast majority of placenta-derived factors are synthesized in the syncytiotrophoblast, which covers all placental villous trees as well as parts of the inner surfaces of chorionic and basal plates [1]. Besides hormones, cytokines, and growth factors, components of the maternal renin-angiotensin system (RAS) are considered as potent key players at the maternal-fetal interface. The activity of maternal RAS is mediated through various ANG peptides and receptors and is suggested to be influenced at various stages of gestation by contribution from tissue-based RAS in the ovaries and the uteroplacental unit [2]. The main effector molecule of RAS is angiotensin II (AngII), which acts on the angiotensin II type 1 receptor (AT1R, encoded by AGTR1) and the AngII type 2 receptor (AT2R, encoded by AGTR2). Both receptors have been described to be expressed in trophoblast subpopulations at the maternal-fetal interface, with AT1R predominantly expressed by differentiated trophoblasts, i.e., the syncytiotrophoblast and extravillous trophoblasts in cell columns, and AT2R expressed in proliferating villous cytotrophoblasts [3]. Immunohistochemistry located AT1R at the microvillous plasma membrane of the syncytiotrophoblast, which enables direct interaction with maternal circulating AngII and, thereby, induction of an endocrine and/or immune-modulating response by the placenta. Application of AngII stimulates secretion of factors such as placental lactogen, estradiol, and the soluble form of vascular endothelial growth factor receptor 1 (sFlt-1) from human placental explants via AT1R [4,5,6].

Besides acting as powerful vasoconstrictor, AngII is suggested to exert a pro-inflammatory effect on leukocytes, endothelial cells, and vascular smooth muscle cells [7]. Since maternal plasma AngII levels are significantly rising from the second trimester of pregnancy onwards [8], it is tempting to speculate about a role of maternal AngII in inducing an inflammatory response in human placenta. AngII functions via AT1R as an inflammatory mediator through activation of the nuclear factor-κB (NF-κB) and activator protein-1 (AP-1) pathways [9,10,11], which, in turn, induce transcription of proinflammatory adhesion molecules, cytokines, and chemokines. Chemokines are categorized into four subfamilies depending on the number and spacing of the first two cysteine residues in a conserved cysteine structural motif [12]. These four subclasses are referred to as C, CC, CXC, and CX3C, where C is a cysteine and X is any amino-acid residue. Among the CX3C subclass, CX3CL1 (also referred to as fractalkine) is synthesized as transmembrane molecule, which can be released as a soluble isoform into circulation by metalloprotease-dependent shedding [13,14]. CX3CL1 has recently attracted attention in the field of reproductive research by regulating adhesion and migration processes in fetal-maternal interaction at different stages of human pregnancy [15]. Furthermore, a growing body of evidence suggests that a number of pregnancy pathologies, including chorioamnionitis [16], diabetic pregnancy [17], and severe early-onset preeclampsia (PE) [18,19], are associated with increased placental CX3CL1 expression. Furthermore, CX3CL1 is elevated in several cardiovascular diseases like diabetes, obesity, and metabolic syndrome that are also risk factors for an adverse pregnancy outcome and promotes platelet activation and vascular dysfunction in congestive heart failure [20,21].

Herein, we describe the expression profiles and correlation of human placental CX3CL1 and AT1R in first trimester gestation, with consideration of the fetal sex, and test the hypothesis whether exogenously applied AngII, which mimics circulating maternal AngII, can deregulate the expression of CX3CL1 in human first trimester placenta. Moreover, adhesion of monocyte cell line THP-1 to CX3CL1-overexpressing trophoblasts as well as effects of recombinant human CX3CL1 on the transcriptome of human primary monocytes were analyzed.

## 2. Results

In order to determine expression dynamics of placental CX3CL1 and AGTR1 during a human first trimester pregnancy, placental tissue from non-smoking, lean (BMI < 25), healthy women (*n* = 45, Table 1) undergoing elective termination of pregnancy was subjected to quantitative gene expression analysis.

**Figure 1:**
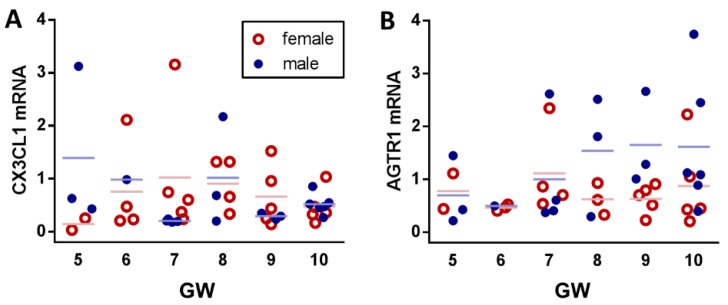
CX3CL1 and AGTR1 mRNA expression in human first trimester placenta. Placental tissue samples (*n* = 45) from healthy, lean (BMI < 25), non-smoking women with gestational ages ranging from 5 weeks to 10 weeks were analyzed for CX3CL1 (**A**) and AGTR1 (**B**) mRNA expression.

Accordingly, both CX3CL1 (Figure 1A) and AGTR1 expression (Figure 1B) revealed no significant changes among different weeks, which suggests stable expression of both genes throughout the first trimester gestation. Moreover, no correlation was found for CX3CL1 and AGTR1 (Pearson, *r* = −0.009, *p* = 0.953) and, in a linear regression model, no influence of maternal and fetal factors could be determined (coefficients were maternal age, BMI, placental volume, fetal crown-rump-length, gestational age). Notably, the fetal sex did not show a remarkable effect on expression dynamics nor correlation of both CX3CL1 and AGTR1. However, for placental CX3CL1, the gestational week 7 (*p* = 0.032) and, for AGTR1, the gestational week 9 (*p* = 0.036) showed sex-dependent differences in expression (Mann-Whitney U, two tailed, alpha = 0.05).

**Figure 2:**
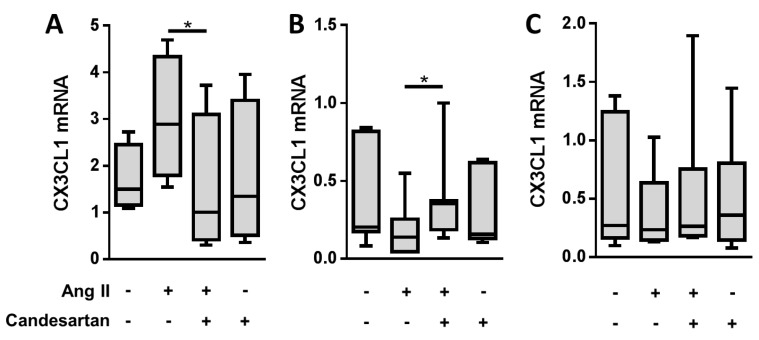
AngII mediates a transient deregulation of placental CX3CL1. Placental explants were cultured with or without AngII (0.1 µM) in the presence or the absence of the AT1R antagonist Candesartan (0.1 µM) for 3 h (**A**), 6 h (**B**), and 24 h (**C**), respectively. Data are presented as median ± IQR (whiskers are min. to max., in **A** *n* = 4, in **B** *n* = 7, in **C** *n* = 7, * *p* ≤ 0.05) from different placental tissues.

Next, we tested the effect of exogenous AngII on placental CX3CL1 expression in human first trimester placental explant culture. qPCR analysis of placental explants showed an initial 1.75-fold upregulation of CX3CL1 expression in response to AngII (0.1 µM) after 3 h (Figure 2A), whereas, after 6 hours, expression was decreased (0.51-fold) when compared to untreated control (Figure 2B). After 24 h, the expression was unchanged (0.95-fold, Figure 2C). Application of the AT1R antagonist candesartan reversed the AngII-mediated deregulation of CX3CL1, while candesartan alone did not show significant effects. Analysis of CX3CL1 expression in placental explants cultured under the same experimental settings for 24 h did not show significant effects of AngII (Figure 2C), which suggests a quick and transient response to the AngII stimulus.

**Figure 3:**
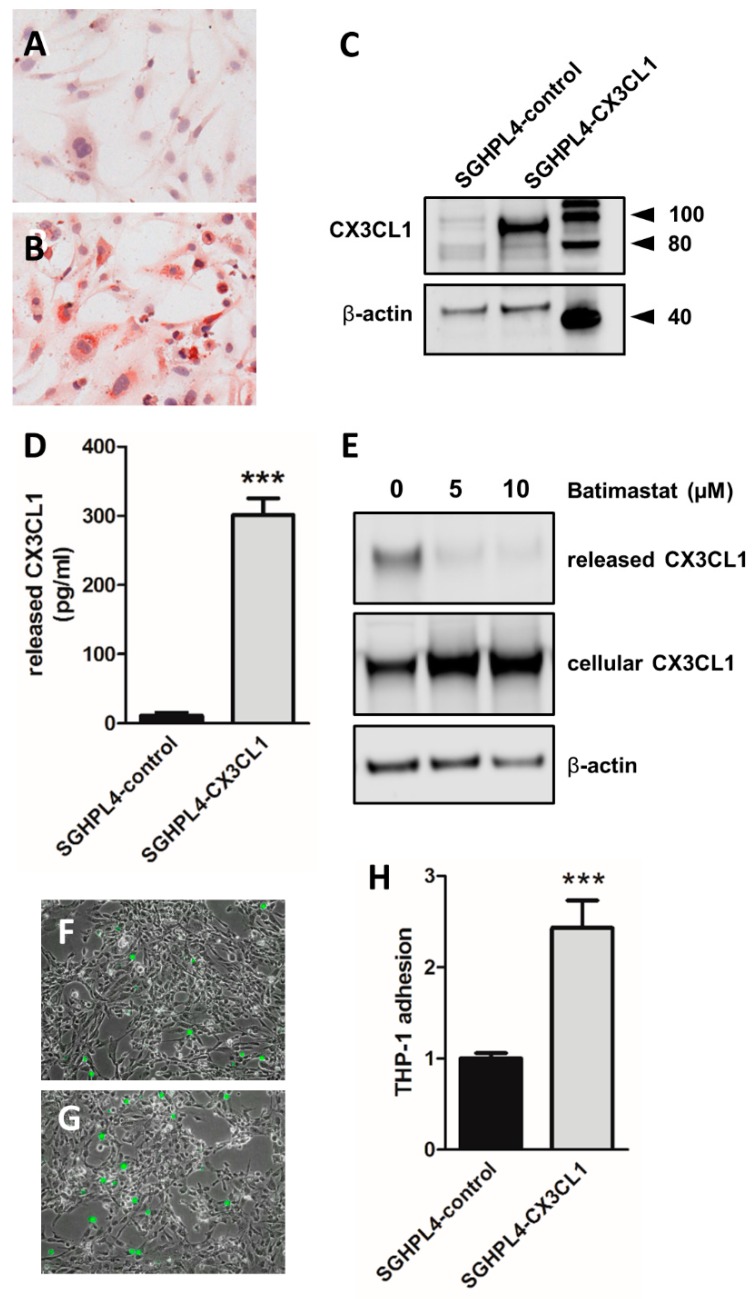
CX3CL1 overexpression in trophoblast cell line SGHPL-4 mediates increased monocyte adherence. SGHPL-4 control cells (**A**) and CX3CL1 stably overexpressing cells (SGHPL-4-CX3CL1, **B**) were stained for CX3CL1 by immunocytochemistry. Western blot (**C**) confirmed CX3CL1-overexpression in SGHPL-4-CX3CL1 cells. ELISA showed abundant release of soluble CX3CL1 in supernatants of SGHPL-4-CX3CL1 after 48 h of culturing (**D**). Metalloprotease inhibitor Batimastat, at concentrations of 5 µM and 10 µM, decreased the release of soluble CX3CL1, while cell associated CX3CL1 accumulated, when compared to the control after 48 h (**E**). Adhesion assays were performed with SGHPL-4 control cells (**F**) and SGHPL-4-CX3CL1 cells (**G**), which were co-cultured with fluorescence CellTracker Green pre-labeled THP-1 monocytes for 90 min. Monocyte adhesion was assessed by acquisition of trophoblast monolayer areas and bound THP-1 cells in phase contrast and green fluorescence channel, respectively. Pixel areas of bound monocytes were related to pixel areas of trophoblast monolayers and data for SGHPL-4-control was set as one. Data are from three independent experiments, using different cell passages and are presented as mean ± SEM *** *p* ≤ 0.001.

Having determined the effect of AngII on placental CX3CL1 expression, we next aimed to analyze the effect of trophoblastic CX3CL1 on the adhesion of monocytes. For this purpose, overexpression of recombinant human CX3CL1 was established in SGHPL-4 cells. While immunocytochemistry for CX3CL1 showed only weak staining of control cells (Figure 3A), CX3CL1-overexpressing cells were distinctly stained (Figure 3B). Immunoblot analysis confirmed immunocytochemistry, which showed a strong band of approximately 95kDa in CX3CL1 overexpressing cells (Figure 3C). Moreover, CX3CL1-overexpressing cells substantially released soluble CX3CL1 (Figure 3D), which was generated in a metalloprotease dependent shedding. Presence of the metalloprotease inhibitor Batimastat, which has previously been shown to effectively block CX3CL1 shedding in placental explants [22], almost completely abolished the release of soluble CX3CL1, while, at the same time, the cellular form accumulated in the cells (Figure 3E). Subsequent adhesion assays revealed a 2.4-fold (*p* < 0.001) increased adhesion of the monocyte cell line THP-1 to CX3CL1-overexpressing SGPHPL4 cells, when compared to control cells (Figure 3F–H).

Since increased monocyte to trophoblast adhesion may contribute to a local pro-inflammatory microenvironment, we next analyzed a panel of 40 cytokines and chemokines released by co-cultures of primary monocytes from healthy, female donors in child-bearing age with either CX3CL1-overexpressing or control SGHPL-4 cells. Among analyzed factors, IL8, CCL19, and CCL13 were significantly increased and an additional 13 analytes showed a trend to increase, when monocytes were co-cultured with CX3CL1-SGHPL-4 cells, compared to controls after 6 h (Table 2).

**Figure 4:**
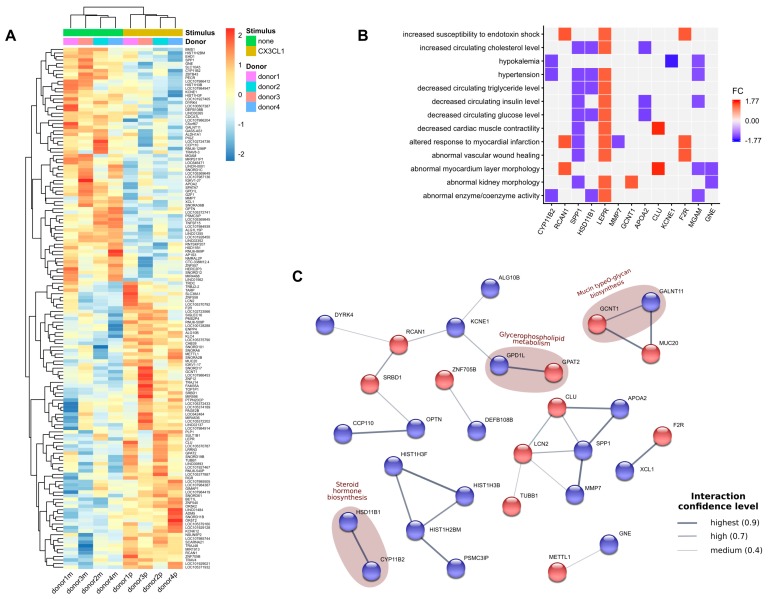
Transcriptome analyses of primary human CD16^+^ monocytes. Primary human monocytes were incubated with (stimulus, brown) or without (none, green) recombinant human CX3CL1. Thereafter, CD16^+^ subtypes were subjected to microarray analysis. The heat map of deregulated genes (**A**), enrichment heatmap for biological processes from Mammalian Phenotype Ontology and Jensen DISEASES Ontology with color representing fold change of a gene (**B**), and protein-protein interaction networks according to STRING database with an edge thickness representing the confidence level of the interaction with upregeulated genes in red and downregulated genes in blue (**C**).

In order to investigate effects of soluble CX3CL1 on monocytes, we next incubated human primary monocytes from healthy, female donors with recombinant human CX3CL1. After incubation, nonclassical (patrolling) and intermediate (pro-inflammatory) CD16^+^ subsets of monocytes, described to express the CX3CL1-receptor CX3CR1 [23,24], were isolated and subjected to microarray analysis of global gene expression profiles. Accordingly, 81 upregulated and 66 downregulated genes were considered differentially expressed (Figure 4A and Figure S1). Among top upregulated genes were clusterin (CLU), lipocalin-2 (LCN2), as well as the leptin receptor (LEPR), whereas top downregulated genes included aldosterone synthase (CYP11B2), osteopontin (SSP1), as well as cortisone reductase (11beta-hydroxysteroid dehydrogenase Type 1, HSD11B1). In addition, approximately 35% of deregulated genes were yet uncharacterized loci and analysis of chromosomal location of deregulated genes revealed two chromosomes (13 and 22) that did not show any deregulated genes (Figure S2). Ontology-based enrichment analysis of the differentially expressed genes revealed a number of processes and phenotypes highly connected to the cardiovascular system such as hypertension or altered response to myocardial infarction (Figure 4B). Lastly, the STRING database derived protein-protein interaction network analysis suggested deregulation of metabolic pathways, including steroid hormone biosynthesis, glycerophospholipid metabolism, and mucin type *O*-glycan biosynthesis in monocytes incubated with recombinant human CX3CL1 (Figure 4C).

## 3. Discussion

The present study suggests that maternal AngII levels affect placental CX3CL1 expression in early human pregnancy. Results from placental explant culture indicate a transient biphasic AT1R-mediated deregulation of placental CX3CL1 expression, including an initial rapid upregulation with a subsequent transient downregulation in response to exogenous AngII. This observation is somehow contrary to previous data obtained in human and murine endothelial as well as vascular smooth muscle cells [25,26]. Higher AngII concentrations and different cell types used in previous studies may in part explain this discrepancy. In this case, we used placental explants, where cells remain in situ, which rather reflects the in vivo situation than cell line monolayers. The lacking association of placental AGTR1 and CX3CL1 in human first trimester, and their stable expression levels independent of maternal factors or fetal sex suggests that the response is most likely not a product of a constitutional increase of receptor expression. Results from previous studies in human umbilical endothelial cells suggest that AngII-induced CX3CL1 synthesis is secondary to TNF-α induction [25]. In line with this assumption, TNF-α strongly induces CX3CL1 expression in human first trimester placenta, and has been suggested as one of the underlying triggers of upregulated CX3CL1 in severe early-onset pre-eclampsia (PE), which is a pathology that is a major contributor to maternal and neonatal morbidity [18,27]. Besides CX3CL1, an activated RAS, including elevated placental AT1R expression, higher AngII sensitivity and autoantibodies against the AT1R (AT1-AA) have been associated with this multifactorial disease [28,29,30,31]. Moreover, oxidative stress, which is generated by an ischemia-reperfusion type phenomenon, is increasingly recognized as another contributing factor of PE [32]. In pregnancy, vasoconstriction is mainly regulated by AngII through AT1R signaling, and ischemia reperfusion type injuries are closely related to the renin-angiotensin-system, which may possibly increase AngII levels after hypoxia-reoxygenation. This also may induce endothelial dysfunction, which is one of the key features of PE [33,34,35]. Furthermore, oxygen fluctuations have been shown to affect placental RAS and AT1R [36,37,38,39], which, in turn, could influence maternal endothelial dysfunction via AT1-AA and augmented vascular AngII sensitivity [40]. Upon such a change in maternal AngII levels, membrane-bound CX3CL1 from endomembrane storage compartments could be rapidly mobilized [41]. This also underlines the possibility of the effects described playing a role in acute CX3CL1 response to fluctuating AngII levels in PE.

Results from the present study confirm the described CX3CL1-mediated monocyte to trophoblast adhesion [42] and additionally suggests that binding of monocytes to CX3CL1-expressing trophoblasts is accompanied by increased secretion of inflammatory cytokines, such as IL-8. This is in line with our recent finding that a disturbed macrophage-trophoblast crosstalk leads to a pro-inflammatory milieu and, thereby, contributes to preeclampsia [43]. Besides analysis of secreted cytokines and chemokines, we performed global transcriptome analysis of primary CD16^+^ monocytes, which showed deregulated expression of immune-modulating factors in response to recombinant human CX3CL1. Among upregulated genes were clusterin, which is an immune modulator described to activate macrophages [24,25], the acute-phase protein lipocalin-2 [26], and the leptin receptor (LEPR). The leptin receptor has been suggested to promote oxidative stress and CD16 expression in human monocytes [27,28]. Among the top downregulated genes were aldosterone synthase (CYP11B2), which is a key enzyme of aldosterone synthesis [29], osteopontin (SSP1), which has been associated with monocyte–macrophage differentiation [30], as well as cortisone reductase (HSD11B1), which generates cortisol from cortisone. Results from transcriptome analysis of CX3CL1-treated primary monocytes and those of trophoblast-monocyte co-culture are, if at all, hardly comparable and it should be stressed at this point that CX3CL1 alone might be insufficient to mediate complete adherence and migration, since a previous study demonstrated that CX3CL1 synergistically acted on monocyte chemotaxis and adhesion in conjunction with other pro-inflammatory cytokines [44]. Whether or not, CX3CL1 overexpression induces expression and release of such synergistic factors or other pro-inflammatory cytokines in trophoblasts remains open and calls for further in depth-analysis. However, CX3CL1-mediated initiation of monocyte adhesion, by orchestrating a pro-inflammatory chemotaxis [45], is suggested to induce a shift of the monocyte population towards the CD16^+^ subset which then predominantly expresses CX3CR1 [46]. Using monocytes from pregnant women would certainly give more insight into pregnancy-related mechanisms, but, to gain CD16^+^ monocyte population in pregnant women, several challenges would have to be met. A paired study design with isolation before and during pregnancy and the difficulty of obtaining informed consent for drawing a fairly big amount of blood required for the monocyte isolation when compared to a routine sampling are some of the biggest difficulties. As an alternative, it would have been interesting to compare our CD16^+^ monocytes from non-pregnant women to maternal CD16^+^ monocytes by using gene expression databases for single cell populations. However, such a database using the Human Cell Atlas (https://www.humancellatlas.org/) with entries about maternal monocytes has yet to be created.

Lastly, our results suggest an impact in biological processes like hypertension, abnormal kidney morphology, myocardial infarction, and myocardium layer morphology. This is in line with the recently described correlation between inflammatory macrophage expansion and vascular remodeling in pulmonary hypertension [47] and could be a possible link to the increased risk of early all-cause mortality of women with a history of preeclampsia that is related to a higher risk for future cardiovascular events and developing end-stage renal disease [48,49]. STRING database network analysis suggest that CX3CL1 deregulates key players involved in glycerophospholipid metabolism and mucin type *O*-glycan biosynthesis. In the process of monocyte to trophoblast adhesion, mucin type o-glycosylation could occur in order to extend the mucin like CX3CL1 stalk, bearing the chemokine domain and to facilitate monocyte binding [50]. Two residues were identified in the CX3CL1 chemokine domain, namely Lys-7 and Arg-47 that are important determinants in mediating the interaction of CX3CL1 with its receptor [51]. The regulation of this metabolic pathway in our microarray analysis concomitant with the regulation of phospholipid synthesis in CX3CL1 treated peripheral monocytes may indicate this mucin stalk extension and, on the other hand, reorganization of the GPCR CX3CR1 on the monocyte cell surface via lipid rafts [52].

## 4. Materials and Methods

### 4.1. Human Specimen

The study was approved by the ethical committee of the Medical University of Graz (26-132 ex 13/14; 15.1.2014). First trimester placental tissues were obtained between week 5 and 12 of gestation with written informed consent from women undergoing legal elective surgical pregnancy terminations. Blood sampling for monocyte isolation was approved by the regional committee of Medical Research Ethics at the Medical Faculty of Charité Berlin (EA4/145/13; 09.1.2014).

### 4.2. qPCR Analysis

Placental tissue was homogenized in RNA Lysis Buffer (peqlab, VWR International, Avantor, Darmstadt, Germany) using an UltraTurrax (IKA) and RNA was isolated, according to the manufacturer’s instructions (peqlab, VWR International, Avantor, Darmstadt, Germany). RNA quality was determined using an Agilent 2100 Bioanalyzer (Agilent Technologies, Santa Clara, CA, USA). Quality check was followed by reverse transcription of 1µg total RNA per reaction using High-Capacity cDNA Reverse Transcription Kit (Applied Biosystems, Foster City, CA, USA), according to the manufacturer’s manual. qPCR was performed with Blue S’Green qPCR Kit (Biozym, Vienna, Austria) using a Bio-Rad CFX96 cycler and specific primers for *CX3CL1* (CX3CL1_For: CACCTTCTGCCATCTGACTGT and CX3CL1_Rev: GCATGATGCCTGGTTCTGTTG), AGTR1 (AGTR1_For: CTATGGAATACCGCTGGCCC and AGTR1_Rev: TGCAGGTGACTTTGGCTACA), and CX3CR1 (CX3CR1_For: CGTCATCAGCATTGATAGGTACCT and CX3CR1_Rev: CTGCACGGTCCGGTTGTT). Fetal sex was determined with specific primers for *DDX3Y* (DDX3Y_For: AGTAGAGGCAACCGGCAGTA and DDX3Y_Rev: TGCACTGGAGTAGGACGAGTA) and for *XIST* (XIST_For: GACACAAGGCCAACGACCTA and XIST_Rev: TCGCTTGGGTCCTCTATCCA). Ct values and relative quantification of gene expression were automatically generated by the CFX Manager 3.1 Software (Bio-Rad Laboratories; Hercules, CA, USA) using the expression of first trimester placenta specific reference genes *YWHAZ* and *CYC1* [53] as a reference.

### 4.3. Placental Explant Culture

Placental villous tissue from human first trimester was thoroughly rinsed in PBS (pH 7.0, 37 °C, Gibco, Life Technologies, Thermo Fisher Scientific, Vienna, Austria) and dissected under a stereoscopic microscope into small pieces of approximately 5 mg moist mass, as described previously [54]. Placental explants were cultured in six well dishes (Nunc) and 4 mL/well DMEM/F12 (1:1, Gibco) supplemented with, penicillin/streptomycin, amphotericin B and L-glutamine, without FCS in a hypoxic workstation (BioSpherix, Redfield, NY, USA) under 2.5% oxygen for indicated time points at 37 °C. For treatments, culture medium was supplemented with Angiotensin II (Sigma-Aldrich, St. Louis, MO, USA) at a working concentration of 0.1 µM. Candesartan (Selleckchem, Munich, Germany) was used at 0.1 µM and DMSO at the same volume served as solvent control.

### 4.4. CX3CL1 Overexpression in SGHPL-4 Cells

Human CX3CL1 coding sequence (sequence ID: NM_002996.4) was amplified by PCR from cDNA with primers containing restriction sites for cloning into a pCAGGS vector (CX3CL1_EcoRI-F: 5′- atc gaattc ATGGCTCCGATATCTCTGTCGT –3′ and CX3CL1_Not1-R: 5′-atc gcggccgc TCACACGGGCACCAGGACATA –3′). PCR reaction was performed with Phusion High-Fidelity DNA Polymerase (New England BioLabs; Ipswich, MA, USA) using 50 ng of cDNA template with 0.2 mM dNTPs, 0.5 µM primers, 3% DMSO in a final volume of 20 µL. Cycling conditions were: 98.0 °C for 30 s, followed by 35 cycles of 98.0 °C for 10 s, 66.0 °C for 30 s, and 72.0 °C for 1.5 min and final extension of 10 min at 72.0 °C. The PCR product was extracted from 1% agarose gel, digested with EcoRI and NotI restriction enzymes, and cloned into the EcoRI/NotI restriction site of the pCAGGS vector, which results in a CX3CL1 expressing construct under the CAGGS promoter (pCAGGS-CX3CL1). To generate a construct for stable transgene overexpression, the CAGGS-CX3CL1 cassette was cut out with SspI restriction enzyme from pCAGGS-CX3CL1 and cloned into EcoRV site, which was generated by site directed mutagenesis into pT2B-puro Sleeping Beauty vector carrying the SV40-puro cassette for puromycin selection. SGHPL-4 cells were electroporated with a 10:1 ratio of vector carrying CX3CL1 overexpression cassette and plasmid containing Sleeping Beauty transposase using a Neon Transfection System (Life Technologies, Carlsbad, CA, USA). Two days post-transfection puromycin selection was carried for two weeks. CX3CL1 overexpressing cells were referred to as SGHPL-4-CX3CL1 and empty vector harboring cells as SGHPL-4-control cells. SGHPL-4 cells were cultured in Ham’s F-10 medium (Merck) and additionally supplemented with 10 % FCS (*v/v*), 100 mg/mL streptomycin, and 100 IU/mL penicillin (Gibco, Life Technologies, Carlsbad, CA, USA).

### 4.5. Culture of Monocyte Cell Line THP-1

The THP-1 cell line was obtained from ECACC and was cultured in RPMI 1640 supplemented with 10% FCS (*v/v*), 100 mg/mL streptomycin, and 100 IU/mL penicillin (Gibco, Life Technologies) at 37 °C in a humidified atmosphere containing 5% CO_2_ in air.

### 4.6. THP-1 Adhesion Assay

THP-1 adhesion experiments were performed as previously described [42]. SGHPL-4-CX3CL1 and SGHPL-4-control cells, respectively, were seeded with a density of 2 × 10^5^ cells/well in 12 well plates. Next day, culture media were replaced by 1 mL RPMI medium containing green fluorescence (CellTracker GreenCMFDA, Life Technologies, Carlsbad, CA, USA) labeled THP-1 cells (4 × 10^5^ cells/well), and cells were co-cultured at 37 °C for 90 min. After co-culture cells were washed with PBS three times and were subsequently fixed with 4% formalin (1 mL/well) for 30 min at RT. THP-1 adhesion was assessed by acquisition of SGHPL-4 monolayer areas (in phase-contrast) and bound THP-1 cells (green fluorescence channel) in a Cell-IQ system (chipman technologies, Tampere, Finland). Pixel areas of bound THP-1 cells were related to pixel areas of trophoblast monolayers in 16 images per well using the Cell-IQ analyzer software (Cell-IQ Analyser v.AN 1.9.0, chipman technologies, Tampere, Finland).

### 4.7. Immunocytochemistry

SGHPL-4 cells were seeded in chamber slides (8 × 10^4^ cells/chamber) and cultured for 48 h. Thereafter, cells were fixed in acetone for 10 minutes and rehydrated in PBS for 5 minutes. Cells were stained using the UltraVision Large Volume Detection System HRP Polymer Kit (Thermo Fisher Scientific, Carlsbad, CA, USA), as previously described [55]. In brief, endogenous peroxidase was blocked with UltraVision hydrogen peroxide block for 10 min. Three washing steps with tris-buffered saline (TBS + Tween) were followed by Ultra Vision Protein Block including 10% human serum for 5 min. Polyclonal goat anti-CX3CL1 (2 μg/mL, AF365, R&D Systems) was diluted in Antibody Diluent (DAKO) and incubated on slides for 45 min at RT. After three TBS washing steps detection was achieved by incubation with HRP-conjugated rabbit anti-goat antibody (5 µg/mL, P0449, Dako, Agilent Technologies), and 3-amino-9-ethylcarbacole (AEC, Thermo Scientific), according to the manufacturer’s instructions. Nuclei were stained with hemalaun and slides were mounted with Kaiser’s glycerol gelatine (Merck). Images were acquired with a Leica microscope (Leica DM6000B) and a digital camera (Olympus DP72, Tokyo, Japan).

### 4.8. Immunoblotting

After incubation, the cells were washed with PBS and lysed in RIPA buffer (Sigma-Aldrich, Saint Louis, MO, USA) including protease inhibitor cocktail (Roche Diagnostics; Mannheim, Germany). Cell lysates were centrifuged at 8000× *g* and 4 °C for 10 min. The concentration of total tissue protein was determined in clear supernatants according to the Lowry method. Additionally, 30 µg total protein were applied to precast 10% Bis-Tris gels (NuPAGE, Novex, Life Technologies). Blotting on a 0.45 µm nitrocellulose membrane (Hybond, Amersham Biosciences, GE Healthcare Life Sciences, Little Chalfont, UK) was followed by analysis of blotting efficiency by Ponceau staining (Ponceau S solution, Sigma Aldrich). Membranes were cut in horizontal strips at molecular weight ranges for target proteins. Goat anti-CX3CL1 antibody (2 μg/mL, R&D Systems) and monoclonal anti-beta actin antibody (12.4 ng/mL, clone AC-15, abcam, Cambridge, UK) were applied to membrane strips overnight at 4 °C. HRP conjugated rabbit anti-goat (1:3000, Dako) and anti-mouse IgG (1:3000, Bio-Rad), respectively, were used as secondary antibodies and incubated on membranes for 2 h at RT. Immunodetection was performed with a chemiluminescent immunodetection kit (Western Bright chemiluminescence Substrate Quantus, Biozym, Austria) according to the manufacturer´s instructions. Images were acquired with FluorChem Q System (Alpha Innotech, Cell Bioscienes, Santa Clara, CA, USA).

### 4.9. Isolation of Human Primary Monocytes

Human primary monocytes were isolated out of 65 ml blood taken from antecubital vein of female donors (*n* = 4), by density gradient centrifugation, using Bicoll (Biochrom) to obtain peripheral blood mononuclear cells (PBMCs), which were subsequently subjected to Pan Monocyte Isolation Kit (130-096-537, MACS, Miltenyi Biotec), according to the manufacturer’s protocol. The mean purity of enriched monocytes was 91.5(±0.9)% and was evaluated on a Canto II multicolor flow cytometry platform (BD). Monocytes were identified after live/dead discrimination and doublet exclusion as being CD3^−^ CD19^−^ CD14^+^ using fluorophore-conjugated monoclonal antibodies (Miltenyi Biotec, Bergisch Gladbach, Germany).

### 4.10. Co-Culture of SGHPL-4 Cells and Human Primary Monocytes

SGHPL-4-CX3CL1 and SGHPL-4-control cells, respectively, were seeded in 2 mL RPMI medium with a density of 4 × 10^6^ cells/well in six-well dishes. The next day, media were replaced by 2 mL RPMI medium containing 6 × 10^5^ human primary monocytes per well. After 1 h of incubation at 37 °C, the cells were gently washed with PBS twice and were cultured in 2 mL RPMI medium including 1% FCS for an additional 6 hours at 37 °C. After co-culturing, the conditioned culture media were collected and centrifuged at 1500× *g* and 4 °C for 5 min. The culture media were stored at −80 °C for subsequent multiplex immunoassays.

### 4.11. Multiplex Immunoassays

Released cytokines and chemokines were analyzed in conditioned culture media using a panel of 40 magnetic bead-based immunoassays (Bio-Plex Pro Human Chemokine Panel, 40-Plex #171-AK99MR2; Bio-Rad), and the Luminex200, according to the manufacturer’s manual.

### 4.12. Stimulation of Human Primary Monocytes with Recombinant Human CX3CL1

Human primary monocytes (5 × 10^6^/mL) were cultured in 5 mL RPMI medium containing 2% FCS in teflon coated bags [56] in the presence or the absence of recombinant human CX3CL1 (50 ng/mL, Biolegend) at 37 °C and 10% CO_2_. After 18 h of culturing, monocytes were collected and subjected to isolation of CD16^+^ monocytes, using the CD16^+^ Monocyte Isolation Kit (Miltenyi Biotec), according to the manufacturer’s manual. The primary monocytes were subjected to a two-step procedure. First, cells were labeled with a non-monocyte depletion cocktail, for labeling and removing CD15^+^ granulocytes and CD56^+^ natural killer cells. Thereafter, the flow through fraction was labeled with CD16^+^ magnetic microbeads for positive selection of CD16^+^ monocytes. CD16 positive selection was performed to enrich CD16^+^ monocytes, described to express the CX3CL1-receptor CX3CR1 [23,24]. CX3CR1 expression in CD16^+^ monocytes was confirmed by qPCR (Figure S3).

### 4.13. Microarray Analysis

Total mRNA was isolated from CD16^+^ monocytes using QIAzol lysis reagent and Qiagen RNeasy mini kit (Qiagen, Hilden, Germany) with on-column deoxyribonuclease I step (Qiagen), according to the manufacturer’s protocol. mRNA quality and concentration was measured by Agilent RNA Kits for the 2100 bioanalyzer. Isolated RNA was converted to DNA and biotin labeled in preparation for transcriptome analysis using the GeneChip^®^ WT PLUS Reagent Kit (Affymetrix, Thermo Fisher Scientific). cDNA was hybridized to Affymetrix HuGene 2.0 ST Array Format 49 Chips, according to the manufacturer’s instructions. Chips were then scanned in the GeneChip^®^ Scanner 3000 7G using Affymetrix GeneChip Command Console software. Microarray data have been deposited in the ArrayExpress database [57] at EMBL-EBI (www.ebi.ac.uk/arrayexpress) under accession number E-MTAB-7484, according to community guidelines, including the “Minimal Information about a Microarray Experiment” (MIAME) [58].

Background subtraction, quantile normalization, and summarization of raw microarray probe intensity values were done with RMA function from the oligo R package. Custom CDF from Brainarray project [59], version 22.0.0, was used to summarize and annotate the probes to Entrez Gene ID. Quality control was done with ArrayQualityMetrics [60] and factoextra (http://www.sthda.com/english/rpkgs/factoextra) R packages. Differential expression analysis was performed with the limma R package [61]. Due to a low sample size, *p*-values < 0.01 were considered as alternative to false discovery rate correction (FDR), according to MAQC/SEQC recommendations [62]. EnrichR R package was used for functional enrichment analysis as well as EnrichR [63] and ShinyGO web services (http://bioinformatics.sdstate.edu/go/). FDR adjusted *p*-value < 0.05 and the top highest EnrichR combined score were used when selecting enriched functional categories. The protein-protein interaction network was extracted from STRING dababase version 11 [64]. Namely, after supplying differentially expressed genes list, we filtered out disconnected nodes and low confidence interactions (< 0.4), which resulted in the network in Figure 4C. Plots were produced with pheatmap (https://cran.r-project.org/web/packages/pheatmap/index.html), karyotypeR [65], EnhancedVolcano (https://doi.org/doi:10.18129/B9.bioc.EnhancedVolcano), and ggplot2 (https://cran.r-project.org/web/packages/ggplot2/index.html) R packages. Genes’ chromosomal locations were obtained with the biomaRt R package [66]. All the computations were done using Bioconductor version 3.6 (BiocInstaller 1.28.0, City, Country) [67] and R version 3.4.4. The source code together with quality control plots is available from a dedicated github repository (https://github.com/sysbio-vo/article-fractalkine-suppl).

### 4.14. Statistical Analysis

Statistical analysis was performed with IBM SPSS Statistics 23 and R 3.5.0. Graphs were created with GraphPad Prism. Obtained data was first analyzed for normal distribution with the Shapiro-Wilk test and the statistically appropriate tests were then chosen, according to the results. Alpha was set at 0.05 and adjusted for multiple testing and alpha-error-accumulation with a Bonferroni correction when needed. For non-parametric analysis of dependent variables, the Friedman’s test was applied as well as the Dunn’s post hoc test, whereas the Pearson correlation coefficient were calculated for normally distributed data. Homogeneity of variances was tested for by Levene’s test and, when appropriate, Welch’s correction was used. A linear regression model was applied in the cohort data for the analysis of possible influencing factors.

## 5. Conclusions

In summary, our data suggest that fluctuations in maternal AngII levels are followed by an immediate upregulation of placental CX3CL1, which, in turn, can contribute to a pro-inflammatory micro-environment by activating patrolling and intermediate monocyte subsets. While the major strength of this study is the use of a high case number of well-characterized human first trimester placental tissues, the use of primary monocytes obtained from non-pregnant women may represent a limitation. Pregnant women were shown to have an increased percentage of nonclassical and intermediate monocytes compared to nonpregnant women, and preeclamptic women had an even higher fraction of CD16^+^ monocytes than pregnant women, which was caused by a selective increase [68]. CX3CL1 promotes CD16 expression of monocytes and their survival [69]. Therefore, our findings support a role of placental CX3CL1 in the pathological increase of intermediate and nonclassical monocytes in preeclamptic women through an AngII mediated mechanism, which is partly responsible for a pro-inflammatory setting.

## Figures and Tables

**Table 1:** Baseline characteristics of the study participants.

Characteristics	Female (*n* = 25)		Male (*n* = 20)		*p* Value
Gestational age (days)	56.84	± 12.62	56.85	± 13.55	0.998
Fetal crown-rump length (CRL)	1.74	± 1.27	1.84	± 1.43	0.797
Placental volume	2.20	± 1.74	1.86	± 0.79	0.625
Maternal age (years)	26.68	± 4.61	25.30	± 5.45	0.362
Maternal weight (kg)	58.32	± 7.40	57.25	± 7.89	0.642
Maternal height (cm)	164.84	± 4.57	165.25	± 6.80	0.811
Maternal BMI	21.44	± 2.34	20.90	± 1.85	0.399

Data are presented as means ± SD.

**Table 2:** Released cytokines and chemokines in co-cultures of SGHPL-4 cells and human primary monocytes.

Cytokine/Chemokine	Aliases	Control (pg/mL)			CX3CL1 (pg/mL)			Fold Change	*p* Value
MIF		5993.0	±	928.5	5740.5	±	653.6	0.96	0.6857
IL8	CXCL8	181.7	±	40.3	347.1	±	23.1	1.91	0.0286
CCL2	MCP-1	37.5	±	6.3	47.3	±	14.6	1.26	0.8857
IL6		26.9	±	7.7	24.1	±	1.5	0.89	1.0000
CCL24	eotaxin-2	18.9	±	12.3	34.9	±	13.5	1.85	0.3005
IL16		16.4	±	3.4	23.7	±	4.1	1.44	0.3429
CCL19	MIP-3β	7.1	±	2.5	14.5	±	1.7	2.03	0.0294
CCL13	MCP-4	3.4	±	0.5	5.8	±	0.3	1.69	0.0286
CXCL16	SCYB16	3.1	±	0.6	3.8	±	0.6	1.25	0.4857
CCL20	MIP-3α	2.8	±	1.2	9.8	±	4.6	3.47	0.3429
CCL3	MIP-1α	1.7	±	0.4	1.8	±	0.1	1.06	0.8857
CCL8	MCP-2	0.9	±	0.2	0.4	±	0.2	0.45	0.3094
CXCL11	I-TAC	0.6	±	0.4	0.8	±	0.3	1.26	0.8794
CXCL5	ENA-78	n.d.			198.3	±	67.5		
CXCL1	Gro-α	n.d.			62.0	±	36.6		
CCL21	6Ckine	n.d.			49.4	±	9.9		
CXCL6	GCP-2	n.d.			13.4	±	7.8		
IL10		n.d.			6.3	±	0.6		
IL1β		n.d.			0.5	±	0.3		

Data are presented as mean ± SEM, *n* = 4, n.d.: not detected, Mann Whitney test.

## Supplementary Materials

Supplementary materials can be found at http://www.mdpi.com/1422-0067/20/3/641/s1.
