# Supplementary material for: Placental CX3CL1 is Deregulated by Angiotensin II and Contributes to a Pro-Inflammatory Trophoblast-Monocyte Interaction
Source: Int J Mol Sci. 2019 Feb 2;20(3):641. doi: 10.3390/ijms20030641 (PMC6387455; doi:10.3390/ijms20030641)
Supplement: Supplementary file 1 [file ijms-20-00641-s001.pdf]

Supplementary Materials

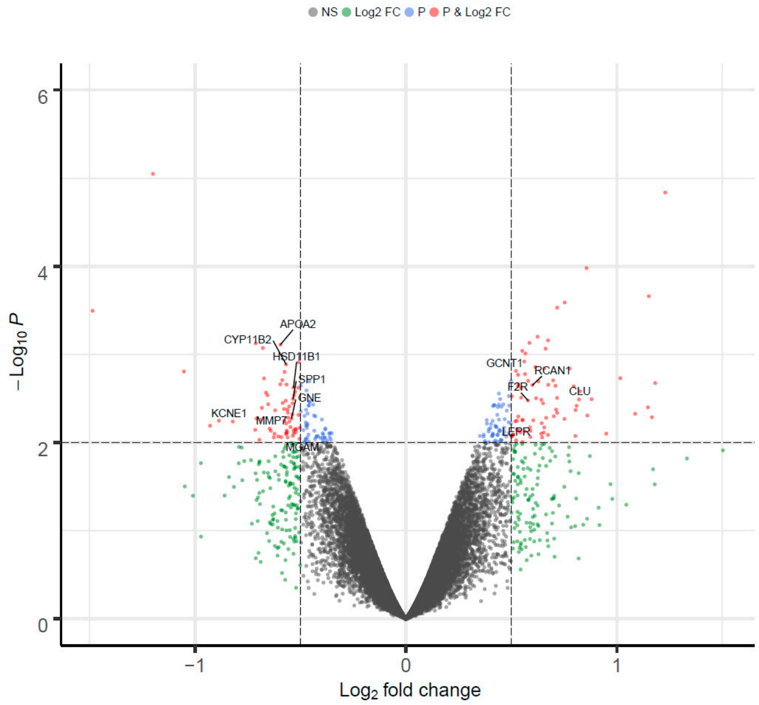

Figure S1. *Volcano plot of differentially expressed genes in CX3CL1-treated CD16*[+] *monocytes*. Red dots represent differentially expressed genes with *p*-valus < 0.01 and logFC > 0.5, blue–genes with logFC < 0.5, green–genes with logFC > 0.5 and *p*-valule > 0.01, while gray dots represent genes considered completely non significant (NS). The labels show differentially expressed genes, which are included in enriched functional and phenotype categories based on MPO and Jensen DISEASES.

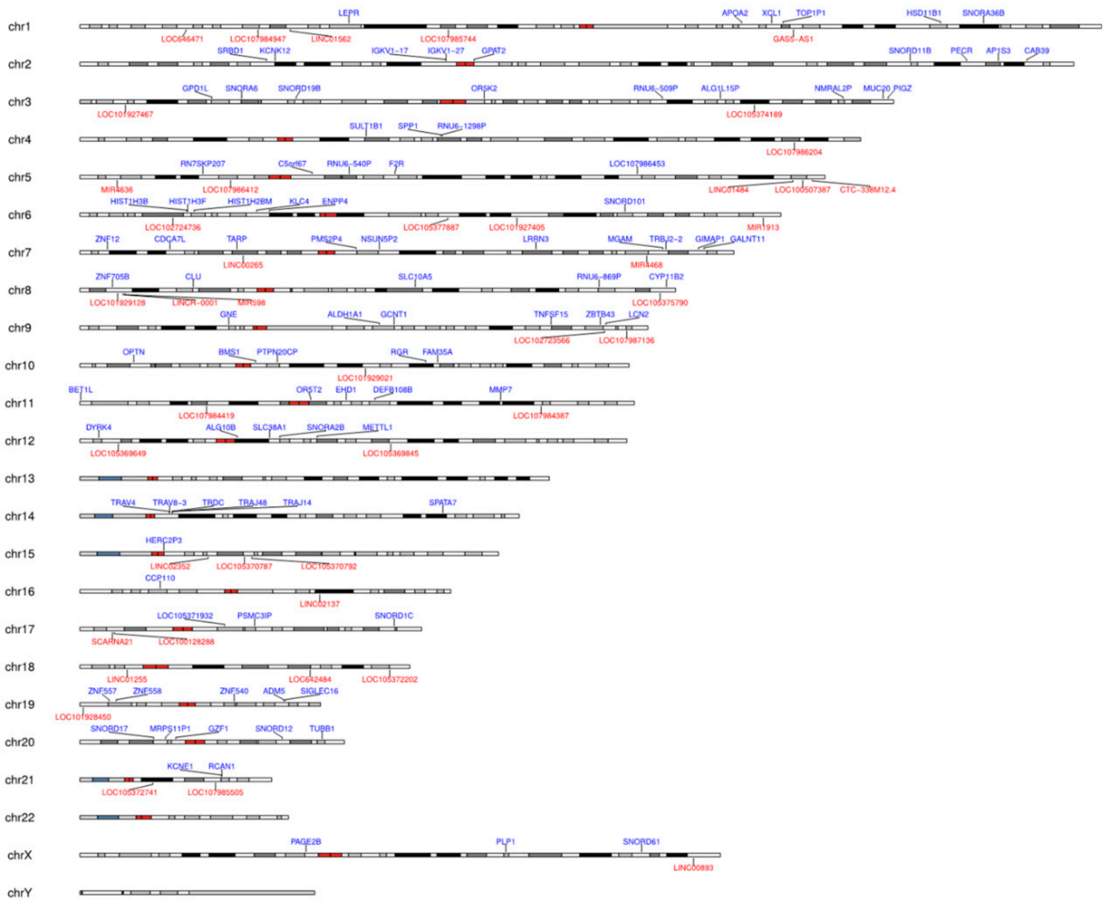

Figure S2. *Chromosomal location of deregulated genes***.** Deregulated genes are shown according to their chromosomal location. Non-coding genes are in red, coding in blue. Chromosomes 13 and 22 did not show deregulated genes.

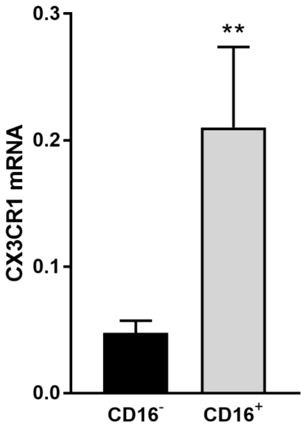

Figure S3. *CX3CR1 expression in monocyte subsets*. CX3CR1 expression was 4.4-fold higher in CD16+, when compared to CD16- monocytes. Data are presented as mean ± SEM from four monocyte preparations ** $p \leq 0.01$.
